# Incidence rates and contemporary trends in primary urethral cancer

**DOI:** 10.1007/s10552-021-01416-2

**Published:** 2021-03-22

**Authors:** Mike Wenzel, Luigi Nocera, Claudia Collà Ruvolo, Christoph Würnschimmel, Zhe Tian, Shahrokh F. Shariat, Fred Saad, Alberto Briganti, Derya Tilki, Philipp Mandel, Andreas Becker, Luis A. Kluth, Felix K. H. Chun, Pierre I. Karakiewicz

**Affiliations:** 1 Department of Urology, University Hospital Frankfurt, Theodor- Stern Kai 7, 60590 Frankfurt am Main, Germany; 2 Cancer Prognostics and Health Outcomes Unit, Division of Urology, University of Montréal Health Center, Montréal, Québec Canada; 3 Department of Urology and Division of Experimental Oncology, URI, Urological Research Institute, IBCAS San Raffaele Scientific Institute, Milan, Italy; 4 Department of Neurosciences, Reproductive Sciences and Odontostomatology, University of Naples Federico II, Naples, Italy; 5 Martini-Klinik Prostate Cancer Center, University Hospital Hamburg-Eppendorf, Hamburg, Germany; 6 Department of Urology, Comprehensive Cancer Center, Medical University of Vienna, Vienna, Austria; 7 Departments of Urology, Weill Cornell Medical College, New York, NY USA; 8 Department of Urology, University of Texas Southwestern, Dallas, TX USA; 9 Department of Urology, Second Faculty of Medicine, Charles University, Prag, Czech Republic; 10 Institute for Urology and Reproductive Health, I.M. Sechenov First Moscow State Medical University, Moscow, Russia; 11 Division of Urology, Department of Special Surgery, Jordan University Hospital, The University of Jordan, Amman, Jordan

**Keywords:** Urethral cancer, Incidence rate, Time trend, Region, Race, Histology

## Abstract

**Purpose:**

We assessed contemporary incidence rates and trends of primary urethral cancer.

**Methods:**

We identified urethral cancer patients within Surveillance, Epidemiology and End Results registry (SEER, 2004–2016). Age-standardized incidence rates per 1,000,000 (ASR) were calculated. Log linear regression analyses were used to compute average annual percent change (AAPC).

**Results:**

From 2004 to 2016, 1907 patients with urethral cancer were diagnosed (ASR 1.69; AAPC: -0.98%, *p* = 0.3). ASR rates were higher in males than in females (2.70 vs. 0.55), respectively and did not change over the time (both *p* = 0.3). Highest incidence rates were recorded in respectively ≥75 (0.77), 55–74 (0.71) and ≤54 (0.19) years of age categories, in that order. African Americans exhibited highest incidence rate (3.33) followed by Caucasians (1.72), other race groups (1.57) and Hispanics (1.57), in that order. A significant decrease occurred over time in Hispanics, but not in other race groups. In African Americans, male and female sex-stratified incidence rates were higher than in any other race group. Urothelial histological subtype exhibited highest incidence rate (0.92), followed by squamous cell carcinoma (0.41), adenocarcinoma (0.29) and other histologies (0.20). In stage stratified analyses, T_1_N_0_M_0_ stage exhibited highest incidence rate. However, it decreased over time (−3.00%, *p* = 0.02) in favor of T_1-4_N_1-2_M_0_ stage (+ 2.11%, *p* = 0.02).

**Conclusion:**

Urethral cancer is rare. Its incidence rates are highest in males, elderly patients, African Americans and in urothelial histological subtype. Most urethral cancer cases are T_1_N_0_M_0_, but over time, the incidence of T_1_N_0_M_0_ decreased in favor of T_1-4_N_1-2_M_0_.

## Introduction

Primary urethral cancer is extremely rare [1–4]. Risk factors for primary urethral cancer are for example recurrent urinary tract infections, chronic irritation through catheterization or sexual transmitted diseases [5–7]. Treatment of urethral cancer depends on its stage at presentation. Usually, surgical treatment is recommended, but also radiation therapy can be applied for organ preservation. In metastatic disease, chemotherapy is recommended^2^. While primary urethral cancer originates from the urethra itself, secondary urethral cancer can be caused by a metastatic spread.

In a study by Swartz et al., relying on 1615 patients with primary urethral cancer identified between 1973 and 2002, urethral cancer annual age adjusted incidence rates were respectively 4.3 and 1.5 per million for men and women in the United States. Moreover, important differences between urethral cancer incidence rates have been investigated in this study with regard to race/ethnicity, age groups, histological subtype and regions [1]. However, urethral cancer incidence rates according to patient and tumor characteristics have not been reassessed since 2002. To test for differences in incidence rates in different patient and tumor characteristic groups across all urethral cancer patients is particularly important, since differences exist within those groups [8, 9]. For example, the most frequent histological subtype in males is urothelial vs. adenocarcinoma in female urethral cancer patients [8]. Moreover, differences also exist between racial/ethnic urethral cancer groups [8, 10].

We addressed these knowledge gap and hypothesized that significant differences in incidence rate of urethral cancer and its trends over time may exist.

## Material and methods

### Study population

In the current study we relied on the Surveillance, Epidemiology and End Results registry (SEER) database (2004–2016) to reassess incidence rates regarding different patient cohorts in primary urethral cancer. The current SEER database samples 34.6% of the United States (US) population and approximates it in demographic composition and cancer incidence [11]. Within the SEER database (2004−2016), we identified patients ≥ 18 years old with histologically confirmed primary urethral cancer (International Classification of Disease for Oncology [ICD-O] site code C68.0). WHO classification was used to define histological subtypes as either urothelial vs. squamous cell carcinoma (SCC), adenocarcinoma or other histology [12]. TNM-stage was used according to the 8th edition of malignant tumors [13]. Race groups were defined as Caucasian vs. African American vs. Hispanic or other race group. Regions were grouped due to low incidence rates and cases within each SEER registries: West (Registries Los Angeles, New Mexico, San-Jose-Monterey, Seattle, California, San Francisco-Oakland, Utah, Alaska, Hawaii) vs. Midwest (Registries Detroit and Iowa) vs. North-East (Registries Connecticut and New Jersey) vs. South (Registries Atlanta, Louisiana, Rural Georgia, Greater Georgia, Kentucky). According to the age stratification by Swartz et al., three modified age categories were defined, namely patients ≤54 years, patients 55–74 years and patients ≥75 years [1]. Unknown histology and unknown racial status patients were excluded. Urethral cancers identified only according to death certificate or at autopsy were also excluded. These selection criteria yielded 1907 assessable urethral cancer patients.

### Statistical analysis

Age-adjusted incidence rates per 1,000,000 based on US year 2000 standard population were calculated (19 age groups, US Bureau of the Census, Current Population Reports, Publication 25-110 [Census P25-1130]) and defined as age-standardized rates (ASR). The latter represented weighted averages of age-specific rates, where weights corresponded to proportions of persons in each-age group of a standard population. Log linear regressions were used to compute average annual percent change (AAPC). Absolute annual cases of newly diagnosed urethral cancer cases in the US were calculated, assuming SEER database is representative of the US population, with calculated incidence rate per specific year multiplied with US population in the corresponding year. All tests were two sided with a level of significance set at *p* < 0.05 and R software environment for statistical computing and graphics (version 3.4.3) was used for all analyses.

## Results

**Fig. 1:**
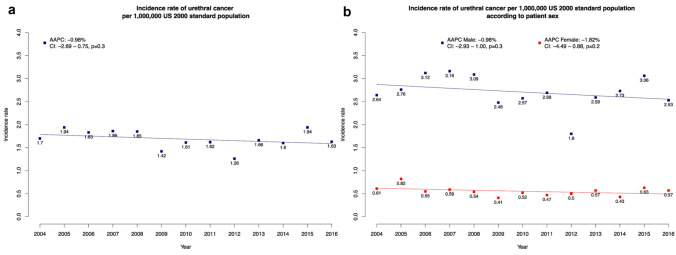
Incidence and trends over time in urethral cancer in the United States. Incidence and trends over time in urethral cancer in the United States from 2004 to 2016, in the entire cohort (**a**) and according to patient sex (**b**). Abbreviations: *AAPC* Average annual percentage changes, *CI* Confidence interval

From 2004 to 2016, 1907 newly diagnosed primary urethral cancers were recorded (Table 1). The overall ASR was 1.69/1,000,000 according to US year 2000 standard population and did not change over the time (AAPC: −0.98%; *p* = 0.3; Fig. 1a). After stratification according to patient sex, overall ASR was higher in males than in females (2.70 vs. 0.55/1,000,000). In temporal trend analyses according to patient sex, ASR in males and females did not change over the time (AAPC: −0.98%, *p* = 0.3 vs. −1.82%, *p* = 0.19; Fig. 1b). In analyses stratified according to patient age, highest ASR was recorded in ≥75 years group (overall ASR: 0.77/1,000,000), followed by 55–74 years (overall ASR: 0.71/1,000,000) and <54 years in that order (overall ASR: 0.19/1,000,000). Absolute numbers of newly diagnosed urethral cancer cases were 436–622 new cases per year between 2004 and 2016 in the US (Table 2).

**Table 1:** Age-standardized incidence rates of urethral cancer

		Age-adjusted incidence rate/1,000,000 US year 2000 standard population			Time trend	
	No of patients (%)	Overall	2004	2016	AAPC	*P* value
Overall	1907 (100)	1.69	1.70	1.63	−0.98%	0.3
Patient sex						
Male	1302 (68.3)	2.70	2.64	2.53	−0.98%	0.3
Female	605 (31.7)	0.55	0.61	0.57	−1.82%	0.19
Race						
Caucasian	1356 (71.1)	1.72	1.71	1.69	−0.32%	0.7
African American	312 (16.4)	3.33	3.01	2.49	−1.90%	0.4
Hispanic	134 (7.0)	1.57	1.59	1.31	−3.33%	0.066
Other	105 (5.5)	1.63	2.03	1.64	−2.81%	0.01
Male race						
Caucasian	1007 (77.3)	2.97	2.99	2.83	−0.35%	0.7
African American	158 (12.1)	5.33	4.45	4.34	−1.86%	0.2
Hispanic	82 (6.3)	2.90	3.53	1.81	−5.96%	< 0.01
Other	55 (4.2)	–	–	–	–	–
Female race						
Caucasian	349 (57.7)	0.92	0.89	1.03	−0.65%	0.6
African American	154 (25.5)	3.15	3.62	2.37	−2.12%	0.3
Hispanic	52 (8.6)	–	–	–	–	–
Other	50 (8.2)	–	–	–	–	–
Histology						
Urothelial	1009 (52.9)	0.94	0.97	0.82	−1.49%	0.14
SCC	455 (23.9)	0.41	0.29	0.49	+ 0.34%	0.8
Adenocarcinoma	278 (14.6)	0.29	9.31	0.24	−0.48%	0.7
Other	165 (8.7)	0.20	0.24	0.26	−0.13%	0.9
Male histology						
Urothelial	835 (64.1)	1.85	2.01	1.57	−1.99%	0.10
SCC	306 (23.5)	0.66	0.54	0.82	+ 0.62%	0.7
Adenocarcinoma	112 (8.6)	0.38	0.31	0.37	−0.19%	0.9
Other	49 (3.8)	–	–	–	–	–
Female histology						
Urothelial	174 (28.8)	0.36	0.34	0.23	−0.89%	0.3
SCC	149 (24.6)	0.36	0.28	0.34	−1.20%	0.3
Adenocarcinoma	166 (27.4)	0.36	0.46	0.29	−1.39%	0.3
Other	116 (19.1)	0.27	0.31	0.37	−0.90%	0.6
Stage						
T1N0M0	596 (31.3)	0.57	0.66	0.39	−3.00%	0.02
T2N0M0	133 (7.0)	0.18	0.14	0.34	+ 1.96%	0.4
T3-4N0M0	516 (27.1)	0.48	0.52	0.31	−1.56%	0.14
T1-4N1-2M0	252 (13.2)	0.26	0.23	0.30	+ 2.11%	0.02
T1-4N0-2M1	181 (9.5)	0.22	0.19	0.19	−0.47%	0.8
Unknown	229 (12.0)	–	–	–	–	–
Male stage						
T1N0M0	429 (32.9)	1.00	1.14	0.61	−3.15%	0.059
T2N0M0	100 (7.7)	0.33	0.27	0.62	+ 1.76%	0.5
T3-4N0M0	347 (26.7)	0.78	0.69	0.44	−1.49%	0.3
T1-4N1-2M0	160 (12.3)	0.44	0.48	0.51	+ 1.26%	0.15
T1-4N0-2M1	117 (9.0)	0.40	0.37	0.36	−0.91%	0.5
Unknown	149 (11.4)	–	–	–	–	–
Age groups						
≤54 years	228 (12.0)	0.19	0.28	0.10	−3.56%	0.11
55–74 years	802 (42.1)	0.71	0.67	0.77	+ 0.21%	0.9
≥75 years	877 (46.0)	0.77	0.70	0.74	−1.27%	0.3
Region						
Midwest	205 (10.7)	2.31	2.39	1.94	−4.10%	0.03
Northeast	320 (16.8)	2.10	2.07	1.53	−2.96%	0.02
South	481 (25.2)	1.99	1.90	1.88	+ 1.77%	0.4
West	901 (47.2)	1.50	1.44	1.75	+ 0.02%	1

Age-standardized incidence rates of urethral cancer and corresponding overall annual percentage changes in 1907 patients, identified within the Surveillance, Epidemiology, and End Results database from 2004 to 2016. Abbreviations: *AAPC* average annual percentage changes, *US* United States, *SCC* Squamous cell carcinoma

**Table 2:** Urethral cancer cases in the United States between 2004 and 2016

	2004	2005	2006	2007	2008	2009	2010	2011	2012	2013	2014	2015	2016
Urethral cancer cases per year in the US	498	573	546	559	564	436	499	504	397	525	508	622	527

Absolute cases of urethral cancer patients per year in the United States (US) during 2004–2016, identified within the Surveillance, Epidemiology, and End Results database and calculated with the incidence rate per 1,000,000 US year 2000 standard population

### The effect of race on ASR

After stratification according to race groups, ASR was highest in African Americans (overall ASR: 3.33/1,000,000), followed by Caucasians (overall ASR: 1.69/1,000,000), other race groups (overall ASR: 1.63/1,000,000) and Hispanics (overall ASR: 1.57/1,000,000). In temporal trend analyses according to race groups, ASR only decreased in other racial group (AAPC: −2.81%; *p* = 0.01).

**Fig. 2:**
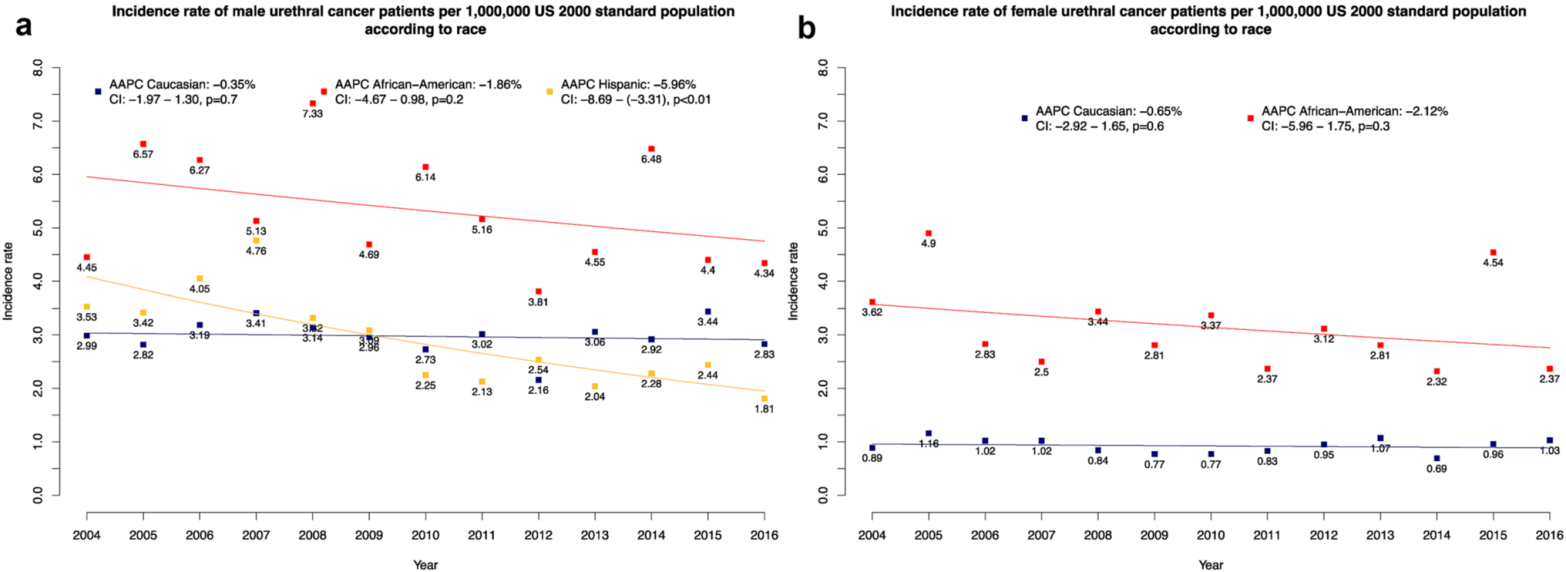
Incidence and trends over time in urethral cancer in the United States from 2004 to 2016, according to race groups and sex for (**a**) male, (**b**) female. Due to sample size limitations, ASR could not be computed for other race group in either males or females. Similarly, sample size limitations prevented computation of incidence of Hispanic females. Abbreviations: *AAPC* Average annual percentage changes, *CI* Confidence interval

After further stratification according to patient sex (Fig. 2a–b), African American males (overall ASR: 5.33/1,000,000) exhibited highest ASR, followed by Caucasians (overall ASR: 1.97/1,000,000) and Hispanics (average ASR: 2.90/1,000,000). In temporal trend analyses, incidence rate of Hispanic males decreased significantly over time (AAPC: −5.96%, *p* < 0.01). In females, African Americans (overall ASR: 3.15/1,000,000) also exhibited higher ASR compared to Caucasians (overall ASR: 0.92/1,000,000). In temporal trend analyses in females that were stratified according to race, no differences were recorded. Due to sample size limitations, ASR could not be computed for other race group in either males or females. Similarly, sample size limitations prevented computation of ASR Hispanic females.

### Effect of histological subtype on ASR

After stratification according to histological subtype, highest ASR was recorded in urothelial histology patients (overall ASR: 0.94/1,000,000), followed by SCC (overall ASR: 0.41/1,000,000), adenocarcinoma (overall ASR: 0.29/1,000,000) and other histology subtype (overall ASR: 0.20/1,000,000).

**Fig. 3:**
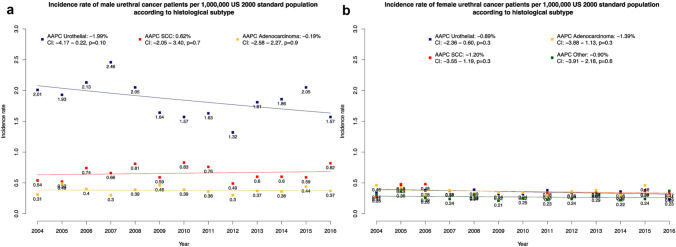
Incidence and trends over time in urethral cancer in the United States from 2004 to 2016, according to histological subtype and sex Male (**a**), female (**b**). Due to sample size limitations, incidence could not be computed for males with other histological subtype. Abbreviations: *AAPC* Average annual percentage changes, *CI* Confidence interval, *SCC* Squamous cell carcinoma

After further stratification according to patient sex (Fig. 3a–b), in males ASR rates were respectively 1.85/1,000,000, 0.66/1,000,000, 0.38/1,000,000 and 0.27/1,000,000 for urothelial, SCC, adenocarcinoma and other histological subtype. Conversely in females, ASR rates were respectively 0.36/1,000,000, 0.36/1,000,000 and 0.36/1,000,000 for urothelial, SCC and adenocarcinoma.

In temporal trend analyses, no clinically meaningful differences were recorded for each of examined histological subtype, with or without further stratification according to patient sex. Due to sample size limitations, ASR could not be computed for males with other histological subtype.

### Effect of region on ASR

After stratification according to SEER regions, highest ASR was recorded in the Midwest (2.31/1,000,000), followed by Northeast (2.10/1,000,000), South (1.99/1,000,000) and West (1.50/1,000,000).

In temporal trend analyses, ASR decreased in the Midwest (AAPC: −4.10%; *p* = 0.03) and Northeast (AAPC: −2.96%; *p* = 0.02). Due to sample size limitations, ASR regional stratification could not be computed according to patient sex.

### Effect of stage at presentation on ASR

**Fig. 4:**
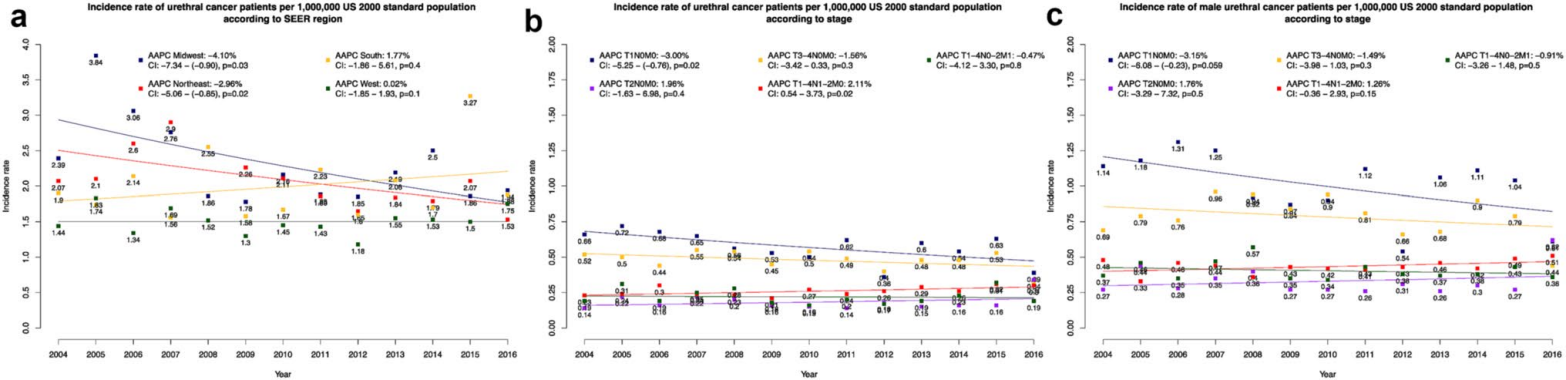
Incidence and trends over time in urethral cancer in the United States from 2004 to 2016, according to SEER region (**a**) and stage at presentation (**b**), as well as stage at presentation in males (**c**) Due to sample size limitations, stratification of regional incidence rates could not be computed according to patient sex, as well as for stage at presentation in females. Abbreviations: *AAPC* Average annual percentage changes, *CI* Confidence interval

After stratification according to stage at presentation (Fig. 4b–c), highest ASR was recorded in T_1_N_0_M_0_ stage (overall ASR: 0.57/1,000,000), followed by T_3-4_N_0_M_0_ (overall ASR: 0.48/1,000,000), T_1-4_N_1-2_M_0_ (overall ASR: 0.26/1,000,000), T_1-4_N_0-2_M_1_ (overall ASR: 0.19/1,000,000) and T_2_N_0_M_0_ (overall ASR: 0.18/1,000,000), in that order. After further stratification according to male sex, ASR rates were respectively recorded 1.00 vs. 0.33 vs. 0.78 vs. 0.44 vs. 0.40 per 1,000,000 for T_1_N_0_M_0_, T_2_N_0_M_0_, T_3-4_N_0_M_0_, T_1-4_N_1-2_M_0_ and T_1-4_N_0-2_M_1_, respectively. In temporal trend analyses, T_1_N_0_M_0_ stage decreased (AAPC: −3.00%; *p* = 0.02) and T_1-4_N_1-2_M_0_ stage increased over time (AAPC: + 2.11%; *p* = 0.03). In temporal trend analyses according to male sex, T_1_N_0_M_0_ stage did not change over time (AAPC: −3.15%, *p* = 0.06). Due to sample size limitations, ASR could not be computed for other stage in females.

## Discussion

We hypothesized that important differences may exist in urethral cancer ASR in contemporary patients, according to gender, age, race, histological subtype, region and stage. We tested this hypothesis within the SEER database 2004–2016 and arrived at several noteworthy observations.

First, we corroborated an important difference in ASR, relative to the previous figures reported by Swartz et al. [1]. Overall, ASR in urethral cancer was 1.69/1,000,000. After stratification according to patient sex, ASR was 2.70 vs. 0.55/1,000,000 in males vs. females, respectively. Moreover, in temporal trend analyses, ASR decreased in both sexes at a similar rate. Although, the absolute ASR is different in contemporary urethral cancer patients, relative to more historic controls, our findings are comparable to Swartz et al., with respect to sex distribution [1]. Specifically, ASR was higher in males than in females. However, comparisons of absolute rates cannot be made, due to methodological and patient population differences.

Second, we identified important differences according to race, as well as patient sex. Specifically, stratification according to race differed between males and females. In consequence, sex-specific results focusing on race were reported. In males, highest ASR was recorded in African Americans, followed by Caucasians and Hispanics, in that order. The same relationship was recorded in females. However, in females, the absolute numbers were lower in African Americans and Caucasians than those reported in males. Finally, ASR could not be computed for Hispanic females due to insufficient numbers. Despite those differences, the temporal trends were highly comparable between males and females, in African American and Caucasian patient groups. Our findings are comparable to Swartz et al., who also reported higher historic incidence rates in African American males and females, relative to Caucasian males and females [1].

Third, we identified important differences according to histological subtypes, as well as according to patient sex. Specifically, stratification according to histological subtypes differed between males and females. In consequence, sex-specific results focusing on histological subtypes were reported. In males, highest incidence rates were recorded in urothelial histological subtype, followed by SCC and adenocarcinoma. In females, incidence rates were comparable between all histological subtype groups. In temporal trend analyses, in both males and females no ASR differences were recorded according to histological subtype. To the best of our knowledge, no contemporary study reported incidence rates according to histological subtype of urethral cancer. In consequence, our data cannot directly be compared to contemporary studies. However, our observations are comparable with previous reports, where urothelial histological subtype was predominant in male urethral cancer patients and were more equal distribution of histological subtype (urothelial, SCC, adenocarcinoma and other) was recorded in female urethral cancer patients [8, 9, 14–18].

Fourth, we tested for differences in ASR according to four SEER regions, namely Midwest, Northeast, South and West. It is of note that important sample size differences exist between those four regions. Specifically, Midwest included 205 observations vs. 901 in West vs. 481 in South vs. 320 in Northeast. Despite numeric and regional membership differences, we corroborated ASR regional differences. These were in agreement with regional differences described by Swartz et al. [1]. Neither Swartz et al. nor the current data can provide firm indications to explain those differences. Moreover, similar to the study by Swartz et al., we also observed the highest incidence of urethral cancer patients in the age category of patients ≥75 years [1]. However, no significant changes were observed in the incidence of all age categories over time. These observations are noteworthy, since with demographic changes an increasing incidence in the oldest age category may have been expected.

Fifth, important differences in stage stratified analyses were recorded. Overall ASR was highest in T_1_N_0_M_0_, followed by T_3-4_N_0_M_0_, T_1-4_N_1-2_M_0_, T_1-4_N_0-2_M_1_ and T_2_N_0_M_0_ stage. After stratification according to male sex, highest ASR was also reported in T_1_N_0_M_0_ stage, followed by T_3-4_N_0_M_0_. Due to limited sample size, incidence rates could not be computed in females. In consequence, the overall rates very closely approximated those recorded in males. In temporal trend analyses, T1N0M0 stage significantly decreased over time (−3.00%, *p* = 0.02) in favor of T1-4N1-2M0 stage (+ 2.11%, *p* = 0.02). In temporal trend analyses that focused on males, the same pattern of stage distribution was observed. To the best of our knowledge, we are the first to report ASR according to stage at presentation in urethral cancer. In consequence, our data cannot be compared to previous investigations. Nonetheless, our findings require consideration in clinical practice, due to the increase in unfavorable stage T_1-4_N_1-2_M_0_ rate.

Taken together, our results provided important observations about urethral cancer incidence and its trends over time. First, urethral cancer ASR is very low, relative to other urologic primaries [19–22]. Its ASR is highest in males, elderly patients, African Americans and in urothelial histological subtype. Most incident cases are stage T_1_N_0_M_0_. However, over time, the importance of T_1_N_0_M_0_ decreased in favor of T_1-4_N_1-2_M_0_. This observation is worrisome and may be indicative in diagnostic delays.

Our work has limitations and should be interpreted in the context of its retrospective and population-based design. Second, our results relied on US population and may not be generalizable to other western countries. Third, our cohort relies on a small sample that resulted in lack of significant differences in some subgroup comparisons. Fourth, histologic diagnoses in the SEER database are derived from medical records, without central review. However, it should be emphasized that the SEER database is designed to providing proportional representation of the United States’ population and only the National Cancer Data Base can provide a larger sample of urethral cancer patients, without providing cancer-specific mortality rates that are required in any cancer analysis.

## Data Availability

All datasets generated for this study are included in the manuscript.

## Code availability

Software R statistics (version 3.6.1).
